# Antimetabolite TTL-315 selectively kills glucose-deprived cancer cells and enhances responses to cytotoxic chemotherapy in preclinical models of cancer

**DOI:** 10.18632/oncotarget.7058

**Published:** 2016-01-28

**Authors:** James DuHadaway, George C. Prendergast

**Affiliations:** ^1^ Lankenau Institute for Medical Research, Wynnewood, PA, USA; ^2^ Sidney Kimmel Cancer Center and Department of Pathology, Anatomy and Cell Biology, Sidney Kimmel Medical College, Thomas Jefferson University, Philadelpia, PA, USA

**Keywords:** cancer metabolism, tumor microenvironment, thiol homeostasis, pentose phosphate cycle, chemotherapy

## Abstract

Maintaining thiol homeostasis is an imperative for cancer cell survival in the nutrient-deprived microenvironment of solid tumors. Despite this metabolic vulnerability, a selective approach has yet to be developed to disrupt thiol homeostasis in solid tumors for therapeutic purposes. In this study, we report the identification of 2-mercaptopropionyl glycine disulfide (TTL-315) as a novel antimetabolite that blocks cell survival in a manner conditional on glucose deprivation. In the presence of glucose, TTL-315 lacks cytotoxic effects in normal cells where it is detoxified by reduction to 2-mercaptopropionyl glycine, a compound with known clinical pharmacologic and safety profiles. In several rodent models of aggressive breast, lung and skin cancers, TTL-315 blocked tumor growth and cooperated with the DNA damaging drug cisplatin to trigger tumor regression. Our results offer preclinical proof of concept for TTL-315 as a novel antimetabolite to help selectively eradicate solid tumors by exploiting the glucose-deprived conditions of the tumor microenvironment.

## INTRODUCTION

Therapeutic strategies to attack common metabolic aberrations in cancer, including the use of antimetabolites, offer appeal because of their potentially broad applications in the treatment of patients with cancer. It is widely appreciated that glycolysis is activated in most solid tumors, but it is less known that the same is true for the oxidative pentose phosphate cycle (OPPC), a metabolic branch in the glycolysis pathway downstream of hexokinase. OPPC is responsible for generating much of the NADPH for which cancer cells require to maintain reduced glutathione at levels sufficient for thiol homeostasis and cell survival [1-5]. Solid tumors the requirement of solid tumors for thiol homeostasis is greater than normal tissues for at least two reasons. First, there is a heavier reliance on cell survival mechanisms in the highly oxidized nutrient-deprived microenvironment of a solid tumor. Glucose depletion occurs commonly in regions of bulky tumors, owing to high glucose metabolism combined with poor perfusion due to a disorganized blood vasculature. Indeed, cancer cells in the solid tumor microenvironment evolve responses to survive the stresses of glucose deprivation which overlaps with hypoxic regions of the tumor to significant extent [6-8]. Glucose deprivation places a great strain on thiol homeostasis in all cells because of the reliance of the OPPC pathway on glucose to produce NADPH, a critical factor in thiol homeostasis. Second, cancer cells rely heavily on DNA repair functions regulated by thiol redox, such as Ku or DNA-PK, which are involved in double strand break repair [4, 5, 9]. Overall, the acute need of cancer cells for glucose/NADPH-dependent thiol homeostasis in the solid tumor microenvironment highlights a general selective metabolic weakness that can be exploited for therapeutic ends.

Recently, new evidence of the vital connection between NADPH production, thiol homeostasis and cancer cell survival under glucose-deprived conditions was illustrated in cell biochemistry studies with the compound hydroxylethyldisulfide (HEDS) [10], a substrate of the glutathione-dependent enzyme thioredoxin reductase [11]. Under normal glucose conditions, HEDS is reduced to Δ-mercaptoethanol (Δ-ME) without toxic effect, but under conditions of glucose deprivation, HEDS adds a stress to thiol homeostasis that was sufficient to trigger p53-independent cell death [10]. Earlier studies of HEDS confirm its relationship with OPPC and thiol homeostasis as a probe of OPPC-dependent thiol oxidative stress and Ku-dependent DNA double strand break repair [3-5, 12], but only recently did it become apparent that HEDS is cytotoxic to cancer cells when glucose is deprived [10].

The need for thiol homeostasis to support DNA repair functions increases interest in dithiol compounds that might leverage the efficacy of DNA damaging therapies including chemotherapy and radiotherapy. Where the interface between thiol homeostasis and metabolism has been investigated in cancer it has mainly been in the context of hypoxia rather than nutrient deprivation. Moreover, strategies to develop selective cancer therapies in this area remain inchoate. Cancer cells located in hypoxic, glucose-deprived regions are well known for their recalcitrance to therapy, comprising a wellspring for stem-like, drug-resistant and pro-metastatic cell populations most often responsible for the demise of cancer patients. Thus, compounds that can sensitize glucose-deprived cancer cells to destruction may offer useful alternatives, alone or as adjuvants for radiotherapy or chemotherapy. In this study, we further develop this concept by offering evidence of the antitumor properties of a novel disulfide compound, TTL-315, which is more potent than similar disulfides studied previously and suited to consider for translation into the clinical oncology setting for patient treatment.

## RESULTS

### TTL-315 is a cytotoxin conditional on glucose deprivation

**Figure 1:**
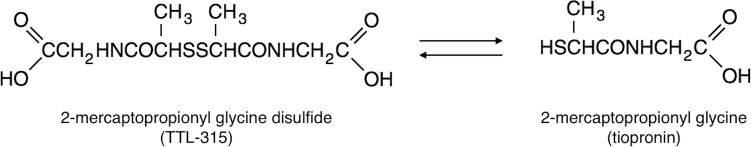
Chemical structure of TTL-315 and its bioreductive relationship with 2-mercaptopropionyl glycine (tiopronin) TTL-315 is the oxidized disulfide of the generic clinical drug tiopronin (2-mercaptopropionyl glycine). Under normative tissue reducing conditions where glucose is available, tiopronin is the bioreductant of TTL-315.

Glucose is a critical nutrient for detoxification of the disulfide compound HEDS which under glucose-deprived conditions *in vitro* triggers cancer cell death [10]. Based on this unique activity, *in vivo* tests of HEDS were explored but this direction was judged impractical due to safety concerns from systemic toxicity of the HEDS bioreductant Δ-ME. In considering other structurally related disulfides with less toxic bioreductive products, we explored the novel compound 2-mercaptopropionyl glycine disulfide (TTL-315), a dimer of the approved clinical drug 2-mercaptopropionyl glycine, tiopronin (also known as thiola), as a potentially safe candidate for analysis (Figure 1).

**Figure 2:**
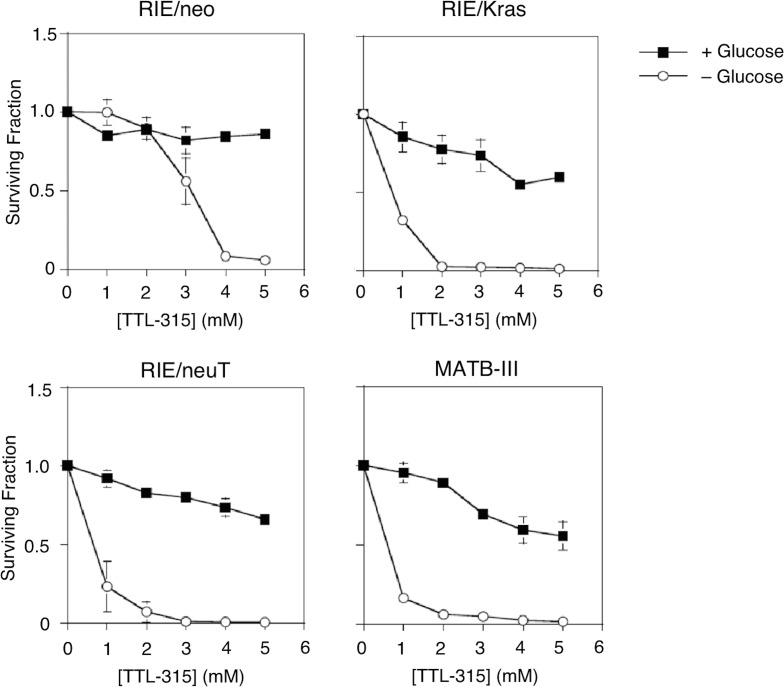
TTL-315 exhibits glucose-dependent cytotoxic properties similar to HEDS Equivalent numbers of cells from the cell population indicated were seeded in growth media and refed the following day with growth media that included or lacked glucose. Four hours later, TTL-315 or vehicle control was added at the concentrations indicated and cells were incubated for 24 hr before analysis as described in the Materials and Methods. The Y-axis presents the relative surviving fraction as a proportion of viable cells counted in replicate cultures (*n* = 3) at the time of TTL-315 or vehicle addition. Data was evaluated by Student's T test.

Following upon studies of HEDS response in colon cancer cells [10], we explored dose responses to TTL-315 in normal and oncogene-transformed variants of the established rat intestinal cell line RIE and in rat MATB-III cells, which are derived from an aggressive mammary carcinoma (Figure 2). The transformed character of the RIE/neuT cells were confirmed by their capability for anchorage-independent growth in soft agar culture (Suppl. Figure 1), as compared to the non-transformed RIE/neo cells and transformed RIE/Kras cells which have been described previously [13]. For experiments investigating TTL-315, equal numbers of cells were seeded into normal growth media and then fed the next day with growth media that included or lacked glucose. Four hours later, TTL-315 or vehicle only was added to the cultures and cells were incubated 24 hr before being subjected to a viability assay that monitors thiol homeostasis [14]. The results presented in Figure 2 show that TTL-315 reduced cell viability unless detoxified by disulfide bioreduction, a condition requiring glucose in the culture media. In the presence of glucose, addition of TTL-315 caused cell growth arrest, whereas in its absence the compound was cytotoxic. Non-transformed RIE/neo cells did not display toxicity to TTL-315 in the presence of glucose, which was also the case to some lesser extent in the transformed RIE/Kras and RIE/neuT cells and the cancer-derived MATB-III cells. However, in the absence of glucose TTL-315 was universally cytotoxic, with the transformed cells exhibiting relatively greater sensitivity. The cytotoxic properties of TTL-315 in glucose-deprived cell cultures was confirmed in other standard cell viability assays (data not shown), arguing against a misleading interpretation of the primary assay. Although further work was needed to fully understand the detoxification reaction, the results suggested that like HEDS itself [10] a latent cytotoxic property of TTL-315 was unmasked in settings of glucose deprivation.

### TTL-315 blocks the growth of tumors and induces tumor regression when combined with cisplatin

**Figure 3:**
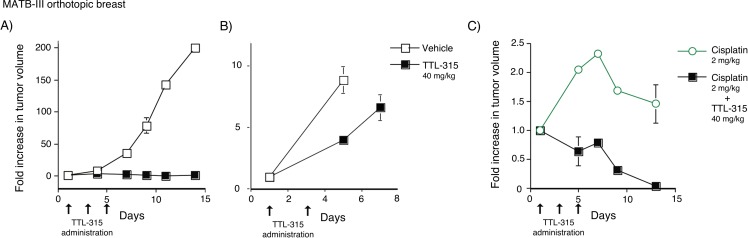
TTL-315 blocks the growth of MATB-III mammary carcinoma and cooperates with cisplatin to trigger regression of bulky established tumors Tumor volumes comparing control and experimental cohorts relative to the starting day of treatment is shown on the Y-axis in all panels. **A.** Prevention design. MATB-III tumor-bearing F344 rats with palpable tumors were administered 40 mg/kg TTL-315 or vehicle only (0.1 ml) by tail vein injection on the days indicated. Mean tumor volume at the start of treatment was 139±4 mm^3^ for mice in the control cohort (*n* = 5) and 127±12 mm^3^ for mice in the experimental cohort (*n* = 5). **B.** Treatment design. Rats with bulky tumors >2000 mm^3^ were treated as before on the days indicated. Mean tumor volume at the start of treatment was 2837±204 mm^3^ for mice in the control cohort (*n* = 5) and 2848±538 mm^3^ for mice in the experimental cohort (*n* = 5). **C.** Cooperation with cisplatin. Rats with bulky tumors were subjected to the treatment design protocol as before except for the addition of cisplatin (2 mg/kg) which was administered at the times indicated to both cohorts. Mean tumor volume at the start of treatment was 2948±180 mm^3^ for mice in the control cohort (cisplatin only) (n=5) and was 2402±218 mm^3^ for mice in the experimental cohort (cisplatin + TTL-315) (*n* = 5). Data was evaluated by Student's T test.

To begin to assess the conditional cytotoxic effects of TTL-315 in the setting of solid tumors, we embarked on a series of experiments in various established preclinical rodent models of breast, lung and skin cancer. MATB-III is an aggressive rat mammary carcinoma the rapid growth of which produces a highly nutrient-deprived tumor microenvironment. In an initial test of the ability of TTL-315 to block tumor growth, we treated MATB-III tumor-bearing mice as tumors became palpable (prevention design). Initial dose and scheduling in pilot experiments ranged empirically. Employing this design, we found that as little as three doses of TTL-315 (40 mg/kg) administered every other day one week after treatment began was sufficient to fully prevent outgrowth of MATB-III tumors (Figure 3A). If tumors were allowed to grow to a bulky size (>2400 mm^3^) before drug administration, TTL-315 adminstered at the same dose slowed but did not block outgrowth (Figure 3B). However, in striking contrast, in bulky tumors where cisplatin chemotherapy was also limited in efficacy, co-administration of TTL-315 at the same dose as before was sufficient to trigger dramatic regressions (Figure 3C).

**Figure 4:**
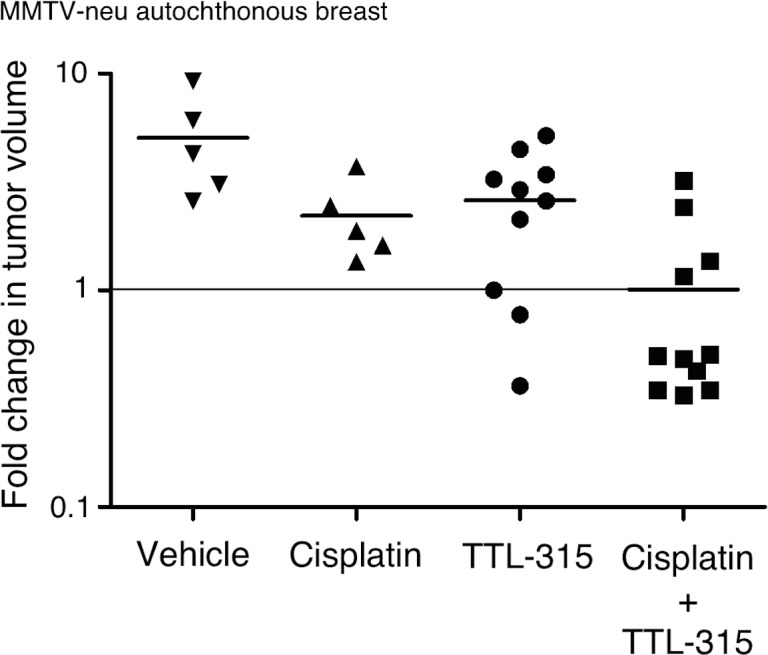
TTL-315 blocks the growth of MMTV-neu mammary carcinoma and cooperates with cisplatin to promote regression of established tumors Tumor volumes comparing control and experimental cohorts relative to the starting day of treatment is shown on a logarithmic scale on the Y-axis. Each dot represents a subject on trial (n). Horizontal bars in each dataset shows the mean of the data. Statistical significance was analyzed by ANOVA. MMTV-neu mice with spontaneously arising palpable mammary tumors (500-1000 mm^3^) were recruited longitudinally to a trial in which TTL-315 (100 mg/kg), cisplatin (1 mg/kg) or vehicle only (0.1 ml volume) was administered by i.p. injection on days 1, 3, 5 of a two-week response trial.

We sought to extend and confirm these observations in a different preclinical model of breast cancer, namely, the transgenic MMTV-neu mouse model of HER2-driven breast carcinoma. Multiparous female MMTV-Neu mice, maintained as previously described [15], present with a high incidence of autochthonous mammary gland carcinomas akin to the human experience in that different secondary ‘hits’, epigenetic pathways and immunoediting events are taken in different individual animals during tumor development. MMTV-neu mice were enrolled randomly into control and experimental treatment groups when tumors reached ∼500-1000 mm^3^. Mice in different cohorts received vehicle only, TTL-315 (100 mg/kg), cisplatin (1 mg/kg) or both drugs on days 1,3,5 of a two-week tumor response assay, a design described in detail in this model for other drug trials elsewhere [16-18]. Two weeks following initiation of the treatment, mice were euthanized for measurement of the final tumor sizes. A comparison of each subject in the cohorts relative to the starting day of treatment confirmed the antitumor activity of TTL-315 and its ability to leverage the efficacy of cisplatin in this model (Figure 4). In this model, TTL-315 was quite potent with 3/10 subjects receiving this agent showed either no tumor growth or frank tumor regression. In combination with cisplatin, which produced no regressions by itself, TTL-315 elicited tumor regressions in 7/11 subjects.

We further explored the antitumor properties of TTL-315 in the established Lewis lung carcinoma model (LLC1), where the potential for cooperation with radiotherapy was of interest based on studies of HEDS as a radiosensitizer [3-5, 19, 20]. In pilot trials, we obtained results from tests of TTL-315 (100 mg/kg) administered i.p. in the LLC1 lung tumor model that were similar to those reported above (Suppl. Figure 2A). LLC1 tumors did not respond to low doses of ionizing radiation tested (2 or 4 Gy), but at 8 Gy which slowed tumor growth the administration of TTL-315 on the same dose and schedule cooperated modestly to attenuate growth further (Suppl. Figure 2B). Overall, the results obtained in three established preclinical models of breast and lung carcinoma that are known for their aggressive properties supported the conclusion that TTL-315 could inhibit tumor growth, enhance the efficacy of DNA damaging therapies and trigger tumor regressions.

### TTL-315 activity does not rely on host immunocompetence or evident systemic toxicity

**Figure 5:**
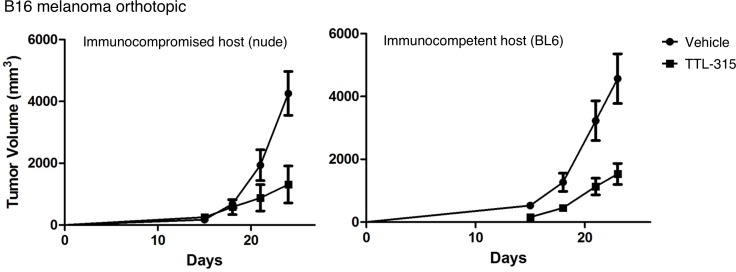
Antitumor properties of TTL-315 do not vary with host immunocompetency C57BL6/J syngeneic hosts (BL6) or immunodeficient nude mice (nu/nu) were injected s.c. with 1 × 10^5^ B16F10 cells (day 0) and enrolled randomly in control or experimental cohorts (*n* = 10 all cohorts). On days 7, 10, 13 mice were administered by i.p. injection vehicle only or 40 mg/kg TTL-315 (0.1 ml total volume). Tumor volumes based on calculations from caliper measurements were determined with an endpoint of 24 days in the experiment. Data was evaluated by Student's T test.

Given emerging evidence of the importance of glucose metabolism on tumor immunity [21, 22], we asked whether the antitumor properties of TTL-315 might be seated in immune alterations. For this direction, we employed B16 murine melanoma based on the importance of immune modulation in melanoma growth and therapeutic response. In this model system, i.p. administration of TTL-315 (40 mg/kg) on a similar schedule at 7, 10, and 13 days after palpable tumor-bearing mice were enrolled yielded tumor growth inhibitions in both immune-competent syngeneic C57BL6 hosts as well as in immune-incompetent nude mouse hosts (Figure 5). While tumor growth differed subtly in each host, no significant differences in the growth inhibition elicted by TTL-315 were apparent. While combinations were not explored in this model, at a first level of investigation it did not appear as though TTL-315 relied upon T cell immunity for its *in vivo* activity, given the similar antitumor effects in these host animals. In a similar vein, we did not obtain evidence of systemic toxicity associated with TTL-315 treatment as a conduit of its antitumor properties, based on observations from exploratory 7 and 28 day toxicity studies in mice (Suppl. Figure 3) and rats (data not shown) or comparisons of the weights of mice treated with TTL-315 at doses of 40-100 mg/kg (single or multiple dosing). These data reinforced the conclusion that TTL-315 is safely tolerated in preclinical models without evidence of gross toxicity on its own or as bioreductively cleared to the monomeric form 2-mercaptopropionyl glycine (tiopronin).

## DISCUSSION

**Figure 6:**
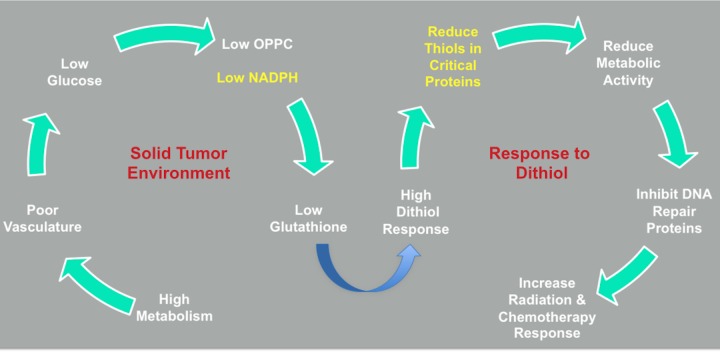
TTL-315 response model TTL-315 is proposed to interfere with thiol redox homeostasis and elicit cell death in glucose-deprived cells where its detoxification is inefficient. A detailed discussion of the bioreductive/detoxification pathways involved have been discussed elsewhere [10]. In the solid tumor microenvironment, the high glucose metabolism combined with a poor blood vasculature leads to glucose deprivation, low OPPC activity and reduced NADPH generation. This situation strains the levels of glutathione needed to preserve thiol homeostasis, which as a result becomes vulnerable to the additional stress created by dithiols that would be reduced by glutathione-dependent redox processes. By intensifying the competition for these processes, thiol redox control of critical cell proteins including DNA repair proteins is compromised. DNA-damaging chemotherapy and radiotherapy provide further strains that cooperate to heighten cellular demise. In attacking this weak point, TTL-315 as a dithiol stressor in glucose-deprived solid tumors may offer additional benefits to cancer management through production of its bioreductive product 2-mercaptopropionyl glycine (tiopronin), as discussed in the text.

The results presented in this study offer an initial preclinical proof of concept for TTL-315 as an antimetabolite with potent antitumor activity. TTL-315 is a disulfide, a thiol dimer, of the approved generic drug tiopronin (aka thiola) which has been used in clinic for many years to treat the disease cystinuria [23, 24]. Bioreduction of TTL-315 yields two monomers of tiopronin, the pharmacology and toxicology for which has been well established clinically. Consistent with the results of preliminary exploratory toxicology conducted in rats and mice, the reduced form of TTL-315, tiopronin, is known to be safe. Therefore, one would expect the safety risks for TTL-315 development to be mitigated to a significant extent. Under normal conditions in healthy tissues, which are provided with sufficient nutrients, TTL-315 is reduced relatively quickly to tiopronin. In contrast, accumulation of TTL-315 in the glucose-deprived microenvironment of a solid tumor, where it is not reduced quickly to tiopronin, would intensify competition for the precious glucose/OPPC/NADPH-dependent thiol-reducing activity which is critical to sustain cancer cell survival in the hostile tumor microenvironment. By stressing a system that is already strained in solid tumors, TTL-315 impairs many local thiol redox-dependent functions, including DNA repair functions. Figure 6 presents a model which illustrates its tumor-selective antimetabolic effects, which are based on glucose insufficiency in solid tumor tissue. The strain imposed by TTL-315 in solid tumors is extended still further by the co-administration of DNA damaging therapies which tax the same thiol redox-dependent functions, suggesting a basis to explain the antitumor cooperativity that was observed in multiple preclinical models of cancer. In summary, our observations are consistent with the interpretation that TTL-315 attacks a metabolic weakness in solid tumors, one which is associated with limited safety risks based on the established safety and pharmacotoxicogical profile of its bioreductant monomer, tiopronin.

NADPH is critically needed to sustain the relatively fragile state of thiol homeostasis in solid tumors. Thus, it is intriguing to consider whether this need may help explain the widespread activation of glycolysis in cancer cells, insofar as the OPPC pathway generating NADPH relies on glucose-6-phosphate synthesized as the first step in glycolysis. As noted above, hypoxic regions of tumors that are notoriously resistant to radiotherapy or chemotherapy are also glucose deprived [8]. Additionally, studies of tissue ischemia indicate that tissue damage occurs as much a result of glucose deprivation as hypoxia [25]. A connection between tissue ischemia, glucose deprivation, and cell death can be understood because under hypoxic conditions NADPH production through the glucose-dependent OPPC pathway is compromised to an extent that protein thiol homeostasis is threatened. The notion that the OPPC pathway is a point of metabolic vulnerability in solid tumors is supported by this study of TTL-315, and extended still further by more detailed cell biochemistry investigations of HEDS indicating that (i.) it is possible to selectively depress NADPH levels in glucose-deprived cancer cells, and (ii.) that this effect sensitizes cancer cells to cytotoxic chemotherapy by compromising thiol-dependent DNA repair proteins [3]. Under normal glucose conditions, TTL-315 is rapidly reduced to its monomeric form tiopronin, eliminating its cytotoxic bioactivity and deactivating its ability to attack a vulnerability only present in the inherently glucose-deprived status of a solid tumor.

As an oxidative cytotoxin, the relative specificity of TTL-315 for glucose-deprived cells seems unique in the literature. As noted elsewhere [10], most chemical oxidants exert their toxicity by general oxidation of lipids, proteins and DNA, lacking specificity for thiols and depleting non-protein thiols in the presence of glucose. Unlike other oxidants, TTL-315 was not expected to be especially toxic to protein or non-protein thiols [3, 4, 19]. Glutathione is the chief non-protein thiol in cells, helping maintain intracellular redox status by scavenging reactive oxygen species or serving as a substrate for enzymes that reduce oxidatively modified proteins, e.g. glutathionylated proteins [3, 19]. It is uncertain as yet the extent to which glutathione redox processes may be affected by TTL-315. However, under conditions of normal glucose levels, cells with normal OPPC activity reduce TTL-315 to tiopronin with little effect on protein and non-protein thiols, much like HEDS to Δ-mercaptoethanol as discussed in detail elsewhere [10].

The production of tiopronin (2-mercaptopropionyl glycine) in normal tissues is an intriguing feature since it may yield additional benefits for cancer treatment. In human cancer cells, this compound exhibits collateral sensitization in relieving multidrug resistance (MDR) through a P-glycoprotein-independent mechanism where glutathione peroxidase has been implicated [26, 27]. Additionally, in a variety of preclinical model systems it has been reported to reduce emesis produced by widely used emetogenic chemotherapies like cisplatin [28-30]. Lastly, evidence has been presented in preclinical models that tiopronin can mitigate chemotherapy-induced liver and kidney toxicity [31-33], probably through antioxidant effects. Although these properties were not examined in the present work, their consideration as useful features of TTL-315 pharmacology offer a further impetus for future study of this unique compound.

## MATERIALS AND METHODS

### Chemicals

TTL-315 was obtained by contract synthesis from Bio-101 and its structure was verified by mass spectroscopy. Stock solutions of each compound were prepared freshly as needed for dilution in cell culture or animal experiments in glucose-free DMEM.

### Cell culture

Rat intestinal epithelial (RIE) cell lines RIE/neo and RIE/Kras were generated and cultured as described previously [13] in Dulbecco's modified Eagle medium (DMEM) supplemented with 5% fetal bovine serum (FBS) and antibiotics. RIE/neuT cells were generated by transformation with a mutant rat neu/Her-2 cDNA (neu-T) expression vector [34]. Cells were passaged 48 hr after transfection in media containing 0.5 mg/ml G418 and drug-resistant cells were pooled to confirm transgene expression by Western analysis along with anchorage-independent growth capability (data not shown). Equal numbers of cells were seeded in multiwell dishes the day before replenishing with normal or glucose-deprived media. TTL-315 or control vehicle (DMSO) was added 4 hr later and viable cell numbers were determined at times indicated by the HEDS-based assay CellCountEZ [14] (Rockland Immunochemicals). Glucose-deprived media was glucose-free DMEM containing 5% FBS and antibiotics. 13762 MATB-III rat mammary adenocarcinoma cells (ATCC) were cultured in McCoy's 5A modified medium supplemented with 10% FBS and antibiotics. LLC-1 murine Lewis lung carcinoma cells (ATCC) and B16F10 murine melanoma cells (ATCC) were cultured in DMEM supplemented with 10% FBS and antibiotics.

### Tumor models and treatments

Established preclinical models of cancer employed in this study included the Fischer rat mammary adenocarcinoma MATB-III, murine Lewis lung carcinoma (LLC-1), murine melanoma B16F10 (B16) and murine MMTV-neu mammary carcinoma. All studies were reviewed and approved by the Institutional Animal Care and Use Committee of the Lankenau Institute for Medical Research and were conducted in a vivarium accredited by the American Association for the Accreditation of Laboratory Animal Care (AAALAC). MATB-III tumors were initiated by orthotopic injection of Fischer 344 rats (Charles River) with 1 × 10^5^ cells suspended in PBS into a mammary fat pad. LLC-1 or B16 tumors were initiated by subcutaneous injection of C57BL6/J mice (Jackson Laboratory) of 1×10^6^ or 1×10^5^ cells suspended in PBS, respectively. The transgenic mouse strain FVB/N-Tg(MMTVneu)202Mul/J abbreviated here as MMTV-neu (Jackson Laboratory) harbors homozygous copies of a rat c-Neu transgene controlled by the mouse mammary tumor virus promoter [35] MMTV-neu mice maintained by in-house husbandry were employed as described in detail elsewhere [16, 36]. Tumor size was determined by caliper measurements. Tumor volumes in the B16 and MMTV-neu models were calculated based on the formula for determining a prolapsed elliptoid (d2_l/0.52) where d is the shorter of the two orthogonal measurements. Tumor volumes in the MATB-III and LLC-1 models were calculated based on caliper measurements in three mutually perpendicular diameters (A,B,C) volume determined as V = (p/6)xAxBxC. For TTL-315 treatments as indicated, tumor-bearing mice with similarly sized tumors were randomized into control and experimental groups before administration of compounds prepared in cell growth media via tail vein injection (MATB-III) or intraperitoneal injection (LLC-1, B16, MMTV-neu).

## SUPPLEMENTARY MATERIAL FIGURES


