# Supplementary material for: Antimetabolite TTL-315 selectively kills glucose-deprived cancer cells and enhances responses to cytotoxic chemotherapy in preclinical models of cancer
Source: Oncotarget. 2016 Jan 28;7(7):7372–80. doi: 10.18632/oncotarget.7058 (PMC4884924; doi:10.18632/oncotarget.7058)
Supplement: Supplementary file 1 [file oncotarget-07-7372-s001.pdf]

Antimetabolite TTL-315 selectively kills glucose-deprived cancer cells and enhances responses to cytotoxic chemotherapy in preclinical models of cancer

Supplementary Material

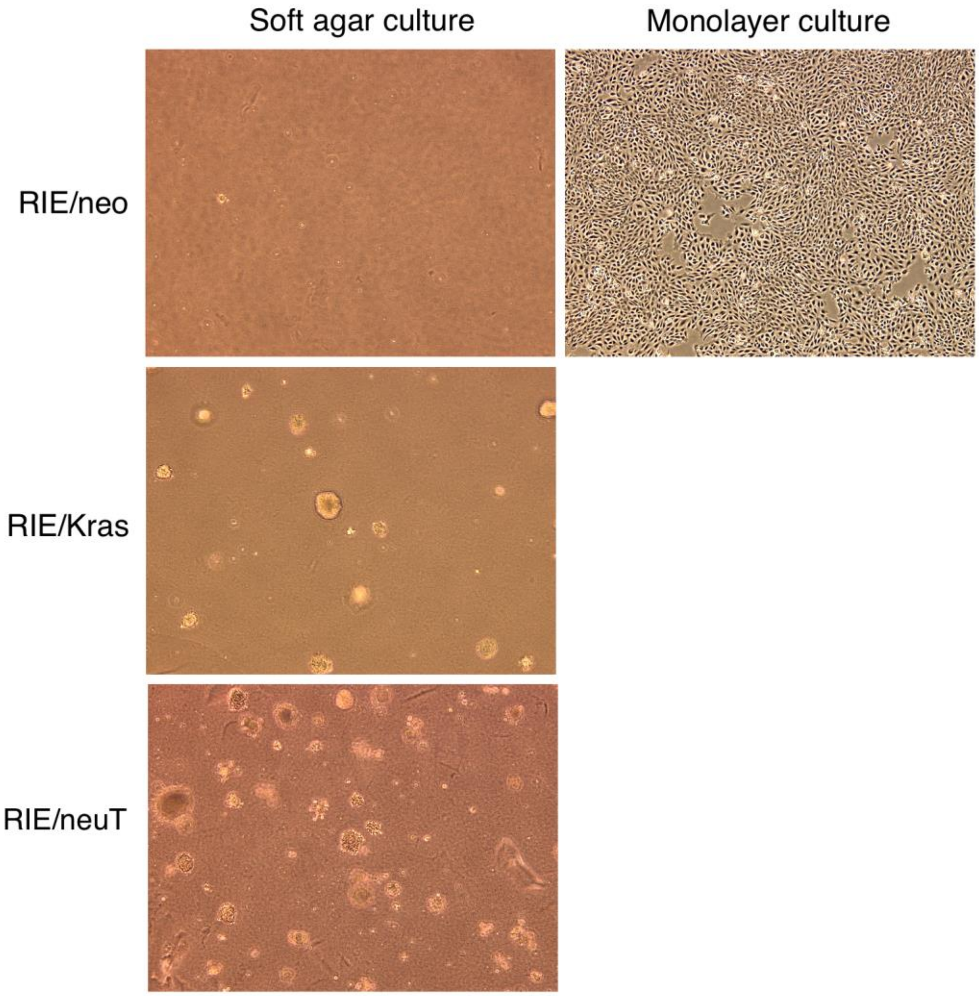

Supplemental Figure 1. Anchorage-independent growth of RIE cell populations. 1 x 10^5 cells of the population indicated were seeded into soft agar cultures as described [13] and cell colonies visualized by light microscopy were photographed 14 days later. Untransformed RIE/neo cells employed as a control cell population seeded into monolayer culture in parallel are shown to confirm the viability of cells seeded into soft agar culture.

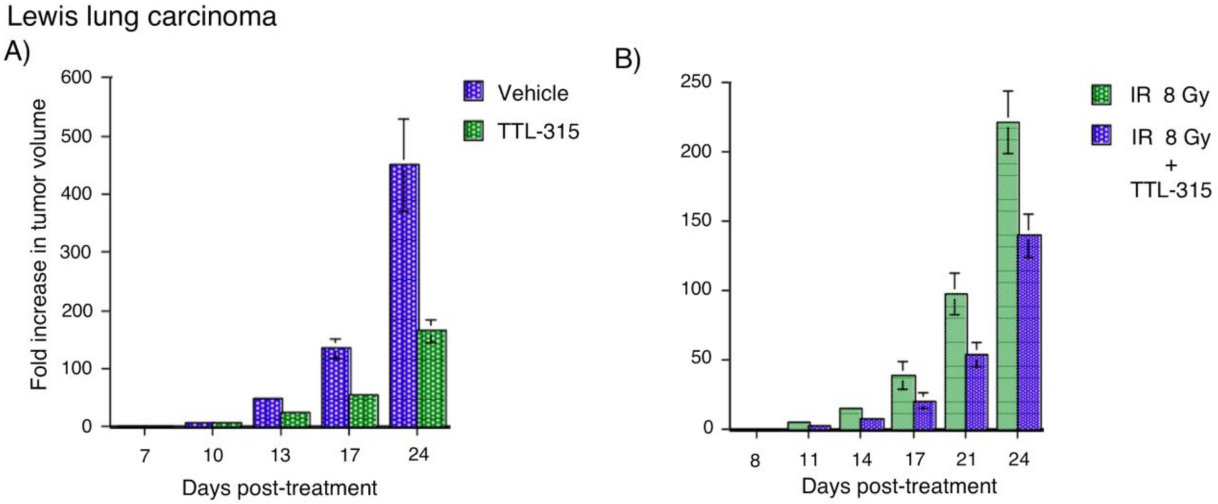

Supplemental Figure 2. TTL-315 inhibits the growth of Lewis lung carcinoma and leverages the growth inhibition elicited by ionizing radiation. Relative tumor volume is shown on the Y-axis in the same manner as in the MATB-III trial shown in Figure 3. (A.) Prevention design. 1 x 10^6 LLC1 cells were injected s.c. into syngeneic C57BL6 mice and treated the next day with TTL-315 (100 mg/kg) on the same three-dose schedule as the MATB-III trial. (B.) Effect on growth inhibiton by ionizing radiation. LLC1 cell-injected mice were treated with TTL-315 or vehicle only as above, except that on day 0 of the treatment mice were also given a single dose of 8 Gy ionizing radiation which slowed the course of tumor growth as compared to the prevention design experiment presented in (A.). Data were analyzed by Student's T test.

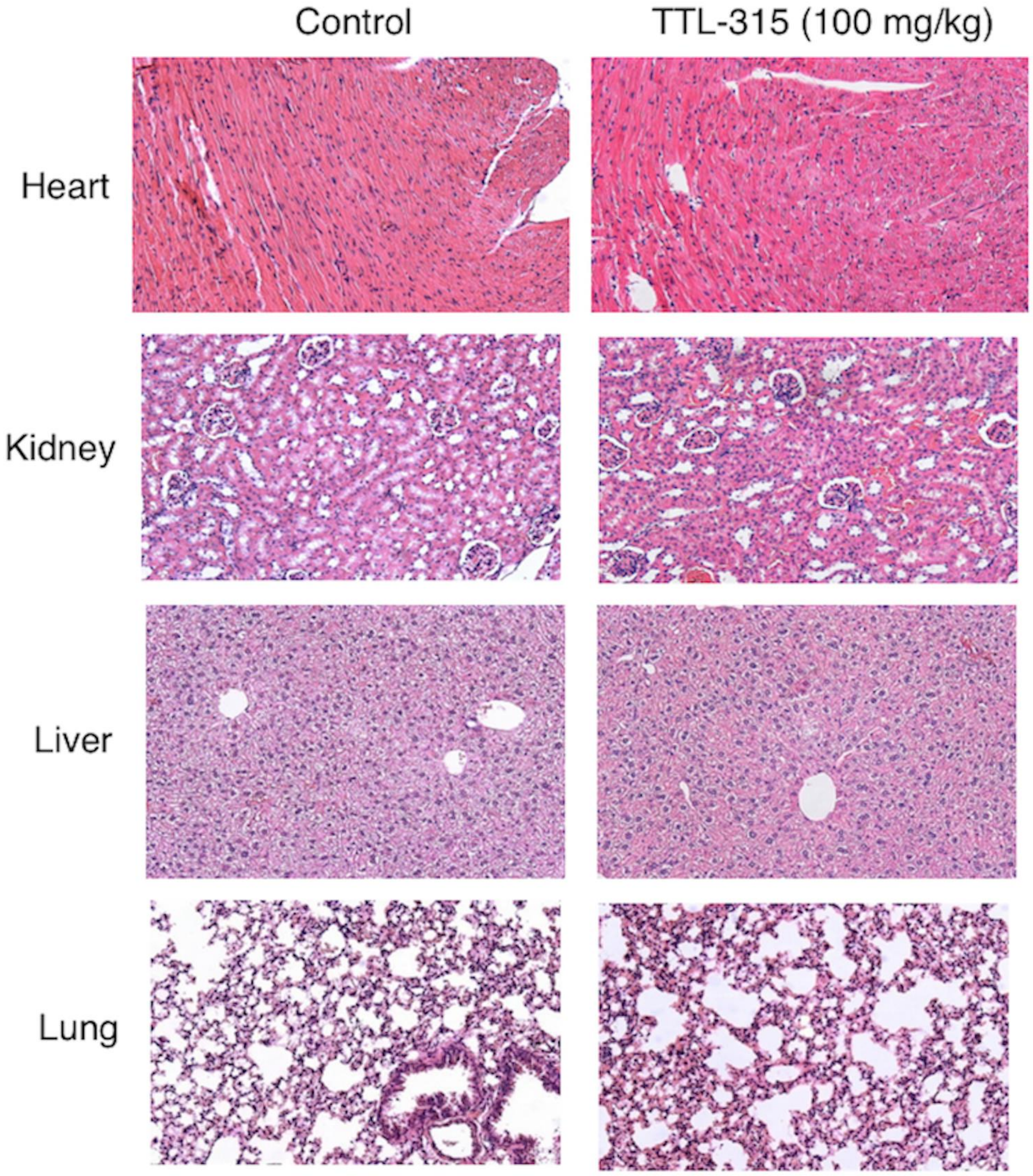

Supplemental Figure 3. Exploratory toxicity of TTL-315 in selected mouse tissues. Results of a traditional 7-day single-dose toxicity study are shown depicting histochemical analysis of selected tissues isolated from normal C57BL6/J mice treated with 100 mg/kg TTL-315 or vehicle only (control).
